# POTENTIAL PREDICTORS OF HEALTH LITERACY IN OLDER ADULTS

**DOI:** 10.1093/geroni/igac059.2248

**Published:** 2022-12-20

**Authors:** Stephanie Rubinstein, Gillian Fennell, Elizabeth Zelinski

**Affiliations:** University of Southern California, Los Angeles, California, United States; University of Southern California, Los Angeles, California, United States; University of Southern California, Los Angeles, California, United States

## Abstract

**Objective:**

Being able to effectively communicate health care needs and understanding health-related information is particularly important as people grow older. In this study, we sought to identify the factors related to subjective health literacy among older adults.

**Methods:**

We examined eight potential predictors of health literacy: subjective memory, cognition, objective health literacy, self-rated health, age, sex, race, education, depression. Our data was derived from a large sample (N=1,272) of participants aged 50 and older who took part in the Health and Retirement Study (HRS; 2008 wave and 2009 internet survey).

**Results:**

Controlling for all other variables, subjective memory (b = .15, p = .02) was positively and self-rated health (b = -.09, p < 0.001) was negatively associated with subjective health literacy. Neither objective health literacy, cognition, nor age were significantly associated with subjective health literacy. Women reported better health literacy than did men (b = .09, p = 0.01). The predictors in this model explained 9% of variation in subjective health literacy. Discussion: These findings may be better understood as we take into consideration the interplay between health literacy, cognition, education, and subjective memory established in extant literature. We discuss the implications of our findings as they relate to healthcare decision-making as well as plans for future research.

